# Supplementary material for: Missed Nursing Care; Prioritizing the Patient’s Needs: An Umbrella Review
Source: Healthcare (Basel). 2024 Jan 16;12(2):224. doi: 10.3390/healthcare12020224 (PMC10815730; doi:10.3390/healthcare12020224)
Supplement: Supplementary file 1 [file healthcare-12-00224-s001.zip › healthcare-2725152-supplementary.pdf]

Author (year)	1	2	3	4	5	6	7	8	9	10	11
Griffiths P, Recio-Saucedo A, Dall'Ora C (2018)	Y	Y	Y	Y	Y	Y	U	Y	Y	Y	Y
Andersson I, Bååth C, Nilsson J, Eklund AJ. A scoping review- Missed nursing (2020)	Y	U	Y	Y	N	Y	U	Y	U	Y	Y
Imam A, Obiesie S, Gathara D (2023)	Y	Y	Y	Y	Y	Y	Y	Y	U	U	N
Duhalde H, Bjuresäter K, Karlsson I (2023)	Y	Y	Y	Y	U	Y	U	Y	N	Y	N
Chiappinotto S, Papastavrou E, Efstathiou G (2022)	Y	Y	Y	Y	U	Y	Y	Y	N	Y	N
Hilario C, Louie-Poon S, Taylor M (2023	Y	Y	Y	U	N/A	Y	N	Y	N	U	Y
Gustafsson N, Leino-Kilpi H, Prga I (2020)	Y	Y	Y	Y	U	Y	Y	Y	U	Y	Y

Stemmer, R., Bassi, E., Ezra (2021)	Y	Y	Y	Y	U	Y	Y	Y	N	U	Y
Suhonen, R., Stolt, M., Habermann, M. (2018)	Y	Y	Y	Y	U	N	N	U	N	N/A	N
Percentage of criteria met (Y)	**100%**	**89%**	**100%**	**89%**	**22%**	**89%**	**44%**	**89%**	**11%**	**67%**	**56%**

Additional file 1: Quality appraisal for included reviews using the Joanna Briggs institute (JBI) tool[19]

Note: JBI tool items: 1. Is the review question clearly and explicitly stated. 2. Were the inclusion criteria appropriate for the review? 3. Was the search strategy appropriate? 4. Were sources and resources used to search for studies adequate? 5. Were the criterial for appraising studies appropriate? 6. Was critical appraisal conducted by two or more reviewers independently? 7. Were there methods to minimise errors in data extraction? 8. Were the methods used to combine studies appropriate? 9. Was the likelihood of publication bias assessed? 10. Were recommendations for policy and/or practice supported by the reported data? 11. Were the specific directives for new research appropriate?
